# Author stance and engagement in research articles on educational equity: A comparison between Chinese and Australian authors

**DOI:** 10.1371/journal.pone.0344083

**Published:** 2026-04-06

**Authors:** Chen Li, Rining Wei

**Affiliations:** 1 School of Foreign Languages, Southeast University, Nanjing, Jiangsu, China; 2 Institute for Language and Cognition, Southeast University, Nanjing, Jiangsu, China; 3 Department of Applied Linguistics, Xi’an Jiaotong-Liverpool University, Suzhou, Jiangsu, China; Bahir Dar University, ETHIOPIA

## Abstract

Author stance and engagement illustrate how the writer interacts with readers in academic texts. Although disciplinary variations in stance-taking and engagement strategies have been extensively documented, scholarly attention has predominantly focused on cross-disciplinary comparisons with limited attention paid to the use of these communicative strategies within specific topical domains. This study aims to examine author stance and engagement in English research articles on educational equity authored by Chinese and Australian authors. Drawing on Hyland’s framework of stance and engagement, it employs a quantitative analysis of interactional metadiscourse markers and engagement devices using the software Wordsmith 4.0 and a log-likelihood calculator. Additionally, the qualitative keyword analysis is conducted to examine other linguistic features that may reflect author stance. The results indicate a statistically significant overuse of stance markers and engagement devices by Chinese authors compared to Australian authors, alongside a more cautious writing style exhibited by Australian authors. Keyword analysis further reveals that Chinese authors tend to adopt a holistic view of educational equity and its relation to different contexts, whereas Australian authors emphasize in-depth research on the topic from diverse perspectives. Cultural interpretations highlight the influence of collective culture versus individualistic culture, analytic vs. holistic thinking, the notion of *xin* (integrity), and conceptions of the self. The research findings can assist authors in recognizing cultural differences across discourse communities and in adopting appropriate stance markers and engagement devices in their academic writing.

## 1. Introduction

Features of author stance and engagementare manifested through the linguistic resources used in the text, which commonly include metadiscourse markers (MDMs), appraisal language resources, and grammatical resources. Extant studies generally focus on identifying the different discourse features across disciplines and languages. However, it remains unclear how linguistic features convey author stance and engagement in research articles on a specific subject, a perspective that could provide deeper insights into the author’s epistemic positions on specific issues.

This study follows Hyland’s framework of stance and engagement, grouping the four subcategories of interactional MDMs under stance, namely hedges, boosters, attitude markers and self-mentions, and distinguishing them from engagement, each with different emphases [1].Author stance concerns the way the author presents himself or herself within the text and conveys his or her opinions, while engagement refers to the strategies through which the author establishes a connection with readers; both contribute to the interactive relationship that defines academic discourse. According to Hyland’s framework, engagement devices include not only engagement markers of reader pronouns and directives, as found in interactional MDMs, but also three additional resources: questions, shared knowledge, and personal asides [1].

Previous studies have primarily focused on author stances and engagement across disciplines and languages. For instance, Hyland’s comparative analysis of MDMs across soft and hard disciplines revealed distinct disciplinary preferences for subjective versus objective styles of discourse [1]. Similarly, Yang’s keyword analysis demonstrated that authors across disciplines place different emphases on various dimensions of the subject matter in discussions [2]. Studies comparing EFL (English as a Foreign Language) speakers with native English speakers have also identified contrasting discursive features [3–5]. In addition to the aforementioned synchronic studies, Xie et al. [6] conducted a diachronic analysis of stance markers in Chinese MA theses and published research articles within the field of applied linguistics from 1991 to 2020, revealing how these stance features evolved over time.

The previous studies have placed greater emphasis on writing style, which concerns how authors present themselves and convey judgements, opinions, and commitments, rather than on “aboutness”, which refers to the actual subject matter of the text. Unfortunately, few studies explore the author’s understanding of the content itself, an aspect that is particularly significant as it may reflect the author’s ideological stance. Although some prior research, such as Yang’s [2] analysis of journal article highlights, has provided insights into authorial stance through keyword analysis, the findings tend to focus more on stylistic features rather than “aboutness”. This is largely because most studies are conducted at the disciplinary level and fail to examine the specific subject matter within the discipline in question. Therefore, scholars’ ideological stances on specific contentious issues, such as educational equity, remain insufficiently examined. Educational equity refers to the equitable distribution of educational resources, enabling diverse actors to participate fully in all levels of society [7]. Given the open-ended nature of such topics, which inherently invite diverse perspectives, this lacuna underscores the need to investigate a broader spectrum of linguistic markers in academic discourse. Doing so can yield more nuanced insights into author stance and engagement with specific subject matter, while also allowing for culturally informed interpretations.

The cultural interpretation of differences in authorial stance and engagement is often framed *solely* through the lens of individualism versus collectivism Previous studies generally agree that individualistic cultures tend to emphasize authorial presence through self-mention and foster a cautious, open-ended rhetorical style by inviting alternative viewpoints and reader participation in the construction of knowledge. In contrast, collectivism tends to avoid personal voice by reducing self-mentions but strengthen the authority in knowledge construction by presenting a unified position without leaving much room for argumentation.

The cultural interpretations based solely upon individualism versus collectivism seem insufficient to account for all subcategories of stance markers and engagement devices. They fall short of offering more nuanced frameworks to understand the influences from various factors when viewed from the *additional* lens of the counterparts of individualism versus collectivism (e.g., analytic vs. holistic thinking, see Section 4.2 for details) d.

Considering the above-mentioned research gaps, the present study raises the following research questions:

(1) What are the differences in author stance and engagement between Chinese and Australian authors when writing English research articles on educational equity?
(2) To what extent can these differences be interpreted through cultural factors?

This study combines interactional MDM analysis and keyword analysis to gain deeper insights into author stance and engagement, providing implications for Chinese authors to strategically project themselves using appropriate stance markers and engagement devices in their English writing. Such strategies enable them to effectively communicate their epistemic positions to target-language readers.

## 2. Literature review

### 2.1. Definitions of author stance and engagement

Author stance refers to the way in which a writer commits to a proposition or expresses an attitude toward the proposition. The engagement denotes the way the author connects to the reader. According to Xu and Nesi [8], there are three primary theories of author stance and engagement: those proposed by Biber, Hyland, and Martin and White. Biber [9] identified seven major linguistic functions, among which the personal and interpersonal functions encompass markers of attitudes toward the communicative event and the relationships between its participants. Martin and White [10] classified appraisal resources into three domains—engagement, attitude, and graduation— to study the speaker’s or writer’s stance and engagement. Hyland’s [11] theory of metadiscourse aligns with the previous two frameworks by recognizing the domains of attitude and engagement. Although all three paradigms focus on the author’s degree of commitment to content and engagement with readers, they propose different frameworks for categorizing the linguistic features of author stance and engagement. For instance, hedges are considered engagement resources in Martin and White’s paradigm, whereas in Hyland’s framework, they are classified differently. We adopt Hyland’s [1] framework because, compared with the theories of Biber, Martin, and White, Hyland’s categorization of interactional MDMs into hedges, boosters, attitude markers, engagement markers, and self-mentions provides a more comprehensive analysis of distinct types of interactional metadiscourse resources.

Table 1 presents the definitions to stance markers.

**Table 1:** Four subcategories of the stance.

Category	Function	Examples
**Hedges**	withhold commitment and open dialogue	might; perhaps; possible; about
**Boosters**	emphasize certainty or close dialogue	in fact; definitely; it is clear that
**Attitude markers**	express writer’s attitude to proposition	unfortunately; I agree; surprisingly
**Self-mentions**	explicit reference to author(s)	I; we; my; me; our

Hedges are linguistic devices that “indicate the writer’s decision to recognize alternative voices and viewpoints and so withhold complete commitment to a proposition” [11]. In contrast, boosters “allow writers to close down alternatives, head off conflicting views and express their certainty in what they say” [11]. While hedges reflect the author’s willingness to acknowledge differing perspectives by presenting information as opinion rather than fact, boosters convey the author’s confidence and certainty regarding the information provided. Consequently, the balance between hedges and boosters “plays an important role in conveying commitment to text content and respect for readers” [11].

Attitude markers express the writer’s “affective, rather than epistemic, attitude to propositions” [11]. Self-mentions refer to “the degree of explicit author presence in the text measured by the frequency of first-person pronouns and possessive adjectives” [11].

In addition to stance markers, engagement devices indicate how the author explicitly addresses readers in the writing to involve them in the discourse and guide them in the interpretation of the writing [11]. The fiver components of engagement devices include both reader pronouns and directives within the scope of interactional MDMs, as well as three other devices: questions, shared knowledge, and personal asides as indicated by Table 2 [1].

**Table 2:** Engagement.

Category	Function	Examples
**Reader pronouns**	bring readers into the discourse explicitly	we; you; your
**Personal asides**	interrupt the argument to address the readers	…both because of his trenchant opinions (often, it is true, insufficiently thought out) and his political opinions.
**Shared knowledge**	ask the reader to identify with particular views	This tendency obviously reflects the preponderance of brand-image advertising in fashion merchandising.
**Directives**	instruct the reader to perform an action	Look at Table 2 again for examples of behavioristic variables.
**Questions**	invite the reader into the conversation	Is it, in fact, necessary to choose between nurture and nature? My contention is that it is not.

Reader pronouns are an explicit way to engage readers in the text, and the inclusive *we* is the most frequently used engagement device in academic writing [1]. Personal asides refer to comments made by the writer that interrupt the argument, creating a brief dialogue with the reader. Shared knowledge is an explicit method of prompting readers to recognize certain views that are considered familiar or widely accepted [1]. Directives guide readers through the text by helping them follow the discussion, perform physical acts, and understand the reasoning [1].

### 2.2. Studies of interactional MDMs in academic publications

Previous research has investigated interactional MDMs across various disciplines. However, as the present study focuses exclusively on texts within the discipline of education, it does not consider cross-disciplinary investigations on interactional MDMs.

More interactional MDMs are observed in academic publications authored by native English authors than in those by non-native authors, regardless of whether the text is written in English or in the author’s native languages [3–5,12]. The following summarizes key findings on the use of interactional MDMs across different subcategories, including hedges, boosters, attitude markers, engagement markers, and self-mentions.

(1) Hedges and boosters

Hedges are used more frequently in US-based international research articles than in Spanish research articles published locally [3]. Similarly, the frequency of hedges is considerably higher in English academic book reviews compared to their Turkish counterparts [12]. Studies of research articles authored by native English authors also indicate a pronounced use of hedges, making them the most frequently employed among all interactional MDMs [13].

Hu and Cao [14] observed a higher frequency of hedges and a lower frequency of boosters in English abstracts published in English-medium journals compared to those published in Chinese-medium journals. A similar pattern was found for English research articles by Anglo-American and Bulgarian authors [15].

(2) Attitude markers

English authors tended to employ more attitude markers than authors in other languages. This difference was observed between English research articles published in international English language journals and Chinese research articles in Chinese-medium journals [5]. A similar trend was also evident in the use of attitude markers [16] in English research articles authored by English and Iranian authors.

(3) Engagement markers

Substantially more engagement markers are used by English authors in English research articles than by Chinese authors in Chinese research articles, and they differ little in using inclusive *we* as an engagement marker [5]. Engagement markers used in Spanish research articles by Spanish authors are slightly more frequent than those in English articles by North American scholars, particularly with respect to the inclusive *we* [3]. However, these differences were not statistically significant. Similary, the frequency of engagement markers in English-medium research articles do not show significant differences between Anglophone and Czech authors though sub-categories of engagement markers tend to show cultural differences [17].

(4) Self-mentions

The frequent use of self-mentions by English authors highlights their authority and credibility in academic writing [13,18]. Most studies have found that English authors employ self-mentions more often than authors writing in other languages [3,19]. This difference is especially pronounced in certain sections of research articles. For instance, in the discussion section of applied linguistics articles, English writers use self-mentions three times more frequently than Iranian writers [20].

### 2.3. Keyword analysis

In addition to examining interactional MDMs, keyword analysis plays a central role in corpus linguistics by exploring the disciplinary cultures and values of academic discourse [21]. It “centres on the qualitative concordance analysis of a set of words which have been identified by a computational procedure as being statistically significant, or ‘key,’ in a specialized corpus, when compared against a larger and more general reference corpus” [21]. The identification of keywords via Wordsmith yields a list of key items specific to the smaller corpus. Moreover, a comparative analysis between two corpora of equal size can be conducted, generating two corresponding keyword lists, with the aim of identifying the words that achieve statistical significance in one corpus relative to the other [22].

Conducting a qualitative concordance analysis of keywords generated from comparing two corpora helps reveal the most significant lexical differences between them in terms of both style and aboutness [22]. A high frequency word would usually indicate the key themes in the discussion and the values embedded in the discourse. If words like *because*, *shall* or *already* are highly frequent, they may disclose more about the style of the writing, referring to the way authors convey opinions and engage with readers [23].

Hyland [18] examined John Swales’ writing style and identifies self-mention, hedging and attitude, reader engagement and considerateness as his rhetorical characteristics. Keyword analysis revealed a high frequency of self-mentions and hedging, which indicated a strong persuasive voice while also showing respect for readers’ differing opinions. Attitude markers were generally positive when evaluating research and work practices.

Groom [21] analyzed phraseological features in two large corpora of book reviews from the disciplines of history and literary criticism, identifying a relationship between phraseology and epistemology in the two corpora through keyword analysis. This study revealed a tendency toward more reiterative phraseologies in literary criticism than in history.

Several metadiscourse studies employing keyword analyses have identified disciplinary variations. Yang [2] analyzed the highlights of research articles across four disciplines—arts and humanities, language and linguistics, engineering and technology, and medicine—to examine their use of evaluative language and interactive discourse. Keyword analysis revealed that “scholars in the soft disciplines tend to highlight divergences and contextualization of the phenomena investigated,” whereas those in the hard sciences “highlight procedures, changes, outcomes or connections, which are more closely associated with the research operations” [2].

According to a wordlist of metadiscursive nouns, the frequent use of word *task* in applied linguistics indicates that scholars in this discipline favor task-based empirical research. Conversely, the frequent use of the word *problem* in electronic engineering suggests that scholars in this discipline are motivated by practical challenges [24]. McGrath and Kuteeva [25] investigated the most frequently occurring words in mathematics research articles and observed that verb forms were preferred over adverbs in the booster category.

The above studies suggest that keyword analysis can more effectively reveal authors’ attitudinal or epistemic positions on specific subject matter. Several studies have adopted only a corpus-based approach to investigate interactional MDMs, resulting in an underutilization of keyword analysis. According to Hyland [13], author stance is not fully covered by the corpus. A wide range of resources is available to illustrate author stance. In addition to the findings from the analysis of interactional MDMs, keyword analysis can contribute to understanding author stance toward the subject matter in a discussion, particularly using keywords and their collocates.

### 2.4 Cultural influences

It is widely accepted that culture plays a significant role in shaping how we organize information, engage with readers, and present ourselves in texts [5]. The metadiscourse employed by authors usually aligns with the expectations of particular cultural and professional communities [26]. Thus, it is valuable to compare the discursive features of different communities using MDMs [26].

Mu et al. [5] argued that the cultural virtues of modesty and respect in Chinese society lead Chinese authors to avoid first-person self-mentions and refrain from overt displays of confidence in academic writing. Li and Xu [19] attributed this reluctance to the collective orientation of Chinese culture. By contrast, English authors, influenced by individualistic ideologies, tend to assert greater authority in academic writing [19].

Regarding the hedging devices identified in English academic writing,Anglo-Saxon scholars tend to favor tentativeness and uncertainty when presenting an argument with a more dialogic style to communicate with readers [3].. In contrast, Confucius’ belief that truth is self-evident without the need for argument may account for the more assertive stance of Chinese authors, leaving little room for alternative perspectives [8,14].

Previous cultural interpretations have tended to focus on Chinese authors’ underuse of self-mentions and English authors’ overuse of hedging devices. However, they have not sufficiently addressed the cultural factors that motivate other linguistic features, such as attitude markers, engagement markers, and certain collocates that reveal author stance and engagement.

## 3. Materials and methods

This study investigated author stance and engagement using a corpus-based approach. First, two corpora of research articles on educational equity were developed. The CHN corpus comprised research articles written by Chinese authors, while the AUS corpus included articles written by Australian authors. Wordsmith 4.0 was used to analyze both interactional MDMs and keywords. The two authors collaboratively identified all interactional MDMs and keywords within their collocations. Subsequently, statistical analysis was conducted to determine significant differences between the two corpora in terms of author stance and engagement. Finally, cultural factors were analyzed to explain the observed differences and their pedagogical implications.

### 3.1. Corpora

The theme of the corpora texts is educational equity, which is widely recognized as an essential issue for human development and is ranked highly in numerous development indices created by international organizations [27]. It is an ideal theme for this study, as it allows for the comparison of different author stances and the use of engagement devices in academic writing due to its contentious nature and significant implications for human development. This study selected 12 English-language research articles on educational equity authored by Chinese scholars for comparative analysis, alongside an equal number of articles on the same topic by Australian authors published in the second decade of the 21st century. In a report entitled *Equity and Quality in Education* by the Organisation for Economic Co-operation and Development (OECD) [28], countries are categorized according to the extent to which student performance is influenced by socioeconomic differences. Australia is the only English-speaking country in the same category as China, where the mean student score is higher than the OECD average, and the relationship between performance and socioeconomic background does not differ significantly from the OECD average. Considering the similarities between the two countries in terms of educational equity, this study created two corpora. The CHN corpus, consisting of 77386 tokens, includes articles written by native Chinese scholars affiliated with Chinese universities and published in international peer-reviewed academic journals. The AUS corpus, comprising 81079 tokens, consists of articles authored by native English speakers affiliated with Australian universities, serving as the counterpart of the CHN corpus. The CHN articles focus on educational equity issues in China, while the AUS articles address educational equity issues in Australia. These articles were drawn from the ProQuest Central database, a growing foundational multidisciplinary resource. In light of the influence of discourse communities on author stance and engagement in academic writing, we ensured that all CHN articles were authored by native Chinese scholars at Chinese universities, and all AUS articles were written by native English speakers at Australian universities, by verifying the authors’ names and institutional affiliations in the published research articles.

### 3.2 Corpus analysis

We adopted a mixed-methods design to collect both quantitative and qualitative data in response to the research questions [29]. Data collection and analysis were conducted using the Wordsmith 4.0 corpus linguistic tool and a log-likelihood calculator (http://ucrel.lancs.ac.uk/llwizard.html). First, we employed the list of interactional MDMs and engagement devices investigated by Hyland [11] to calculate frequencies in the CHN and AUS corpora, respectively. Next, concordances were examined to exclude any items that did not conform to the definitions of interactional MDMs and engagement devices. This task was completed by one of the two co-authors. To ensure accuracy, the other co-author independently repeated the procedure and coded a random sample comprising approximately 10% concordance. In the sample with 366 concordances, the two co-authors agreed on approximately 89% of the items from which MDMs were identified. Any inconsistencies between the two co-authors were discussed, and a consensus was reached for all identified MDMs, resulting in acceptable inter-coder reliability. Subsequently, the log-likelihood calculator was used to compare the frequency counts of MDMs and determine statistically significant differences in the use of the investigated items across the two corpora. Finally, a keyword analysis was performed using Wordsmith 4.0 to further explore features of author stance.

Keyword analysis is typically performed by comparing the target corpus with a larger, more general reference corpus to determine a list of words that are statistically significant. However, this study compared the CHN and AUS corpora to identify keywords that were either overused or underused in one corpus relative to the other. Concordances involving keywords were examined and compared across the CHN and AUS. The terms “overuse”/ “underuse” only indicate that interactional MDMs occur significantly more/less frequently in one corpus compared with the other; they do not imply better/worse usage or users.

## 4. Results and discussion

### 4.1. Differences in author stance and engagement between CHN and AUS

#### 4.1.1. Analysis of stance markers and engagement devices.

Table 3 presents the frequencies of the interactional MDMs in the four stance subcategories.

**Table 3:** Frequency of interactional MDMs of the stance.

	CHN		AUS		Log- Likelihood	
Subcategories of Interactional MDMs	Tokens	Per 1000 words	Tokens	Per 1000 words	LL Value	*p* Value
**Hedges**	473	6.11	278	3.43	+60.72	<.0001^****^
**Boosters**	185	2.39	90	1.11	+38.08	<.0001^****^
**Attitude markers**	135	1.74	96	1.18	+8.56	<.01^**^
**Self-mentions**	97	1.25	216	2.66	−41.02	<.0001^****^

Note. *p < .05, **p < .01, ***p < .001, ****p < .0001.

Significantly more interactive MDMs are observed in CHN than in AUS. Compared to AUS, CHN exhibits an overuse of interactional MDMs in all four subcategories except self-mentions. All log-likelihood values exceed the critical value of 15.13, except for those of attitude markers. According to Rayson et al. [30], this indicates statistically significant differences at *p* < 0.0001, except for attitude markers, which show significance at *p* < 0.01.

(1) Hedge markers

One of the most notable differences is the overuse of hedge markers in CHN compared to AUS, which contrasts with most previous research reporting higher hedge frequencies in academic publications or student essays by native English authors than by non-native authors [3,12,14,15]. Among all hedge markers, *should* exhibits the highest frequency in CHN (94 occurrences), whereas *may* is the most frequently used in AUS (26 occurrences). This marked difference in frequency may be attributed to the frequent use of *should* for making suggestions in the conclusion sections of CHN research articles. Of the 94 occurrences of *should* in CHN, 45 (47.9%) appear in the concluding sections. In contrast, Australian authors employ a variety of ways to make suggestions besides *should*, such as *suggest, need to* and *it is hoped that*. The word *should* appears only 16 times in the AUS, with just two occurrences in the conclusion of the research articles. For example:

a. Meanwhile, the local government should distribute funding and public resources fairly among kindergartens with different sponsoring bodies, and thus create a fair environment for the different types of kindergarten to compete and survive. (CHN)
b. First of all, regular rotation exchange system of teachers should be conducted. (AUS)

(2) Boosters

*Show* is the most frequently used booster in CHN, appearing 76 times (41.08%), whereas it occurrs only 21 times (23.33%) in AUS. The lower frequency of boosters in AUS compared to CHN supports previous research findings that boosters are usually underused in research articles by English-speaking authors compared to those by authors writing in other languages [14,15]. For example:

c. The analysis shows that the factor loading of the measurement index is statistically significant, and the loading of most measurement indexes reaches 0.5. (CHN)
d. Survey data collected showed an extraordinary bias toward sport and personal development, health, and physical education (PDH/PE) in both time allocation and structural support and Principal B’s school had a higher emphasis on sport. (AUS)

Yang [31] emphasized that *show* objective is to present information that can be directly observed. Words such as *indicate* and *suggest* are more subjective because they involve the author’s interpretation; therefore, they may be less convincing. The predominant use of *show* as the most frequent booster in the CHN demonstrates the greater assertiveness of Chinese authors compared to Australian authors.

Knowledge-making practices in academic research typically rely on objective observations of empirical data, which accounts for the frequent use of *show*, *find* and verbs as boosters [32]. However, the second most frequently used booster in CHN is the adverb *clear*, 19 (10.27%), illustrating linguistic variety of boosters.

Effective academic writing tends to favor caution over certainty, as reflected in the relative proportions of hedge markers and boosters [3,11,13]. The ratio of hedge markers to boosters in CHN and AUS is 2.56 and 3.09 respectively, demonstrating a comparatively higher level of writing by Australian authors. According to the diachronic study by Xie et al. [6], the ratio in English research articles in the field of applied linguistics increased first but decreased substantially later to 2.86, which is still higher than that of CHN in this study.

(3) Attitude markers

Contrary to the findings of Mu et al. [5] and Abdollahzadeh [16], there were more attitude markers for the CHN corpus than in the AUS corpus. *Important* and *even* were the two most frequent markers in both corpora. Combined, *important*, *importantly* and *even* accounted for 61.48% of the CHN cases and 55.21% of the AUS cases. *Even* was statistically significantly overused in CHN. In addition to the comparative structure of *even X* (comparative structure), the use of *even* in other forms express the author’s attitude, such as *even though*, *even if*, or *even* alone as an adverb, was also examined. Because *even* emphasizes something unexpected or surprising, the overuse of *even* in CHN indicates a stronger emotional inclination among Chinese authors compared to Australian authors. For example:

e. Universities may need to conduct deeper reforms of internal management, curriculums and teaching, to make even greater efforts to inculcate a spirit of innovation. (CHN)
f. Although this measured and deliberate approach was clearly time consuming, it has resulted in these schools reporting a distinct connection with, and even a loyalty to, the programme. (AUS)

(4) Self-mentions

Chinese authors tended to use significantly fewer self-mentions than Australian authors. In CHN, self-mentions were dominated by exclusive *we* (82.47%) and *our* (13.40%). Similarly, in AUS, exclusive *we* (55.56%) and *our* (27.78%) were the two most frequently used self-mentions. In previous studies, *I* and *my* were usually the most frequent self-mentions in academic writing by native English speakers [19]. However, in this study, *I* is rarely used as a self-mention. This is likely because 75% of all research articles from CHN and AUS were co-authored. For example:

g. First, we adopt a broader concept of SE, which is also known as shadow education. (CHN)
h. However, at the core of our motivation was our passionate desire to provide a specialised teacher-education programme that addressed what we saw as the core issue: the inequitable distribution of our highest performing graduates to more affluent schools. (AUS)

(5) Engagement

Hyland [1] proposed five devices for reader engagement: reader pronouns, personal asides, shared knowledge, directives, and questions. The frequencies of the engagement devices identified in this study are presented in Table 4. According to the log-likelihood results, these devices were significantly overused in CHN compared to AUS.

**Table 4:** Engagement.

	CHN		AUS		Log- Likelihood	
Subcategories of engagement devices	Tokens	Per 1000 words	Tokens	Per 1000 words	LL Value	*p* Value
**Reader pronouns**	24	0.31	3	0.04	+19.59	<.0001^****^
**Personal asides**	26	0.34	26	0.32	+0.03	>.05
**Shared knowledge**	3	0.04	0	0	+4.30	<.05*
**Directives**	20	0.26	9	0.11	+4.81	<.05*
**Questions**	4	0.05	3	0.04	+0.19	> 0.05
**Total**	77	1.00	41	0.51	+12.9	<.001^***^

Note. *p < .05, **p < .01, ***p < .001, ****p < .0001.

In the directives subcategory, both Chinese and Australian authors rarely employ imperatives to engage readers. Among the 20 directives in CHN, only five are imperative uses of *take as (an) example*, while the other 15 are obligation modals of *should*. In AUS, there are only three occurrences of imperatives: *consider* and *note*;and six occurrences of obligation modals: *must*, *need to* and *should*.

Imperatives are generally regarded as face-threatening [33]. In English-medium research articles, obligation modals are used even less frequently than imperatives as engagement markers by both Anglophone and Czech scholars, largely due to their stronger imposition on the reader [17]. In contrast, both Chinese and Australian authors tend to employ obligation modals more often than imperatives for engagement purposes. Furthermore, a significantly higher number of directives are found in articles by Chinese authors compared to those by their Australian counterparts. For example:

i. At the same time, attention should be paid to the needs of the common people for high-quality educational resources. (CHN)
j. For gender equity to be achieved, we need to recognise the value of the roles of everyone outside of their paid employment. (AUS)

#### 4.1.2. Keyword analysis.

Keyword analysis is used to examine words that are overused and underused in one corpus compared to another. The keyword list generated from the CHN and AUS wordlists contain a total of 265 words. This study further investigates these keywords and their collocates within the corpora, finding that the keywords *equality* in CHN, *equity* and *policy* in AUS, reveal significant differences in author stances between Chinese and Australian authors.

(1) Equality and equity

While *equality* and *equity* are often considered synonymous, they carry distinct connotations. *Equity* refers to “being fair, impartial, even handed” [34], whereas *equality* denotes “the right of different groups of people to have a similar social position and receive the same treatment” [35]. The overuse of *equality* (95 occurrences) in the CHN suggests an emphasis on ensuring equal treatment for all individuals in education. Conversely, the overuse of *equity* in AUS (219 occurrences) indicates a concern for fairness tailored to the differing needs of various groups of participants in education. These divergent word choices in research articles by Chinese and Australian authors informed readers of their different stances on this research issue. For example:

k. Promoting educational equality is one of the most important reasons for governments to expand higher education by making it more accessible to the underprivileged. (CHN)
l. This study has highlighted the need for a clear, transparent process for the administration of gender equity policies, including claims for Dependent Care Support expenses. (AUS)

Further investigation into the collocates of *equality* and *equity* found that the most frequently used collocates were *educational equality* and *gender equity* in CHN and AUS, respectively. The second most frequently used collocates reveal author stance features in the two corpora. There are 29 collocates of (*the*) *equality of/in X* in CHN and 37 collocates of *the X of equity* in AUS.

The collocates of *the equality of/in X* primarily highlight areas where issues of educational equality arise, such as *the equality of gender and race*, *the equality of the allocation of educational resources*, *the equality in college admissions*, *the equality in educational opportunities*. For example:

m. Therefore, the equality of compulsory education should not only be reflected on its equal opportunities of school entering, but also its independence of family background. (CHN)

This pattern is similar to one of the most frequent semantic sequences for *of* in studies by Groom [21], which demonstrates CONCEPTUALIZATION + of + PHENOMENON. Chinese authors tend to conceptualize equality issues in the context of education.

In AUS, the collocates of *the X of equity* focus on understanding equity from multiple perspectives, including *the concepts of equity*, *the problems of equity*, *notions of equity*, *the definitions of equity*, *the discourses of equity*, *the redefinition of equity*, *the interpretation of equity*. For example:

n. These are the redefinition of equity, increased marketisation and greater centralisation of decision-making. (AUS)

The different word choices of the Chinese and Australian authors may reflect varying perspectives on educational equity. Chinese authors appear to regard the issue as systemic, often conceptualizing multiple factors in their writing to analyze their correlations within this complex system. In contrast, Australian authors generally focus on examining different aspects of the concept of equity, placing less emphasis on the social impacts of educational equity across different areas.

(2) Policy

The frequency of *policy* is 348 in the AUS group and 134 in the CHN group. The overuse of *policy* in AUS demonstrates its pivotal role in Australian research articles. Australian authors show a strong interest in comprehensive analyses of policy from multiple perspectives, as reflected by collocates such as *policy texts*, *policy documents*, *policy instruments*, *policy tools*, *policy rationales*, *policy logic*, *policy actors*, *policymakers*, *policy problems*, *policy implementation*, *policy development*, *policy emphasis*, *policy framing*, *policy assumptions*, *policy debates*, and *policy priorities*.

However, the collocates of *policy* in the CHN are less diverse than those in AUS, including *policy texts*, *policy document*, *policy design*, *policy implementation*, *policy implementers*, *policy environment*, *policy features*, and *policy objective*.

Although *policy* in both the AUS and CHN is generally interpreted as guidelines to be implemented, Australian authors tend to explore the deeper structure and meaning of policy in their research from different perspectives, such as treating policy as a tool, analyzing its logic and rationales, and understanding its development, emphasis, and framing. In contrast, only a few collocates of *policy* in the CHN focus on meanings beyond implementation, such as *policy design*, *policy environment*, *policy features*, and *policy objectives*.

### 4.2. Cultural interpretations

Many of the research results discussed above can be interpreted in light of the different cultural backgrounds of Chinese and Australian authors, which shape their stances and engagement in writing.

#### 4.2.1. Individualism vs. collectivism.

The most influential framework for understanding cultural values distinguishes between individualism and collectivism [36,37]. Individualism emphasizes “the independence from groups/collectives,” whereas the collectivism highlights “the interdependence of individuals” [38]. According to Hofstede [36], Chinese culture is predominantly collectivist, featuring a *we* consciousness, identity, and belongingness based on collectivist systems; emotional dependence; private lives and opinions influenced by organizations; benefits and duties provided by organizations; the pursuit of prestige and friendships within stable social relationships; and a belief in group-based decisions and particularism. In contrast, many Western countries, including English-speaking countries, belong to individualistic cultures, defined by an *I* consciousness, personal identity, initiative and achievement, emotional independence, private lives and opinions, autonomy and variety, the need for personal enjoyment and specific friendships, and a belief in individual decisions and universal principles.

The holistic perspective and emphasis on interconnected systems of multiple factors revealed in the writing of Chinese authors can be understood as emerging from a dynamic interplay between the cultural values of collectivism and the evolving conventions of academic discourse, each continuously shaping and reinforcing the other. The emotional dependence of individuals on organizations in collectivist cultures may account for the frequent overuse of attitude markers, as the emotional ties connecting individuals and organizations encourage Chinese authors to express and share their emotions in their writing. Similarly, the high frequency of the collocate (*the*) *equality of/in X* in CHN establishes a connection between the issue of educational equity and various contexts.

However, influenced by their individualistic culture, Australian authors are more likely to focus on understanding key concepts and examining how individual factors influence others. The emphasis on the individual contributes to the prevalence of in-depth research on specific concepts from multiple perspectives, as evidenced by the rich collocates of *equity* and *policy*.

#### 4.2.2. Analytic thinking vs. holistic Thinking.

The Western analytic thinking generally focuses on the central object, identifying its attributes and assigning it to a category. However, the Eastern holistic thinking emphasizes the object within its context, the relationships among objects, and the connection between the object and its surrounding context [39,40].

When Chinese authors address the issue of educational equality, they consider the various contexts in which it arises. Conversely, the divergent collates of *policy* in the AUS demonstrate the identification of attributes from multiple perspectives for further analysis of their rationale, problem, emphasis, framing, and so forth, none of which takes the relevant context into account. This distinction can be partly attributed to the low-context culture of most Western societies, where individuals’ attributes are considered independent of their circumstances or relationships. [41].

Westerners typically focus on salient objects and their attributes, while Easterners emphasize the continuities of the objects’ substances and relationships in the environment [41]. For instance, equality is objective because it relates to the distribution of resources. However, equity involves the ethical judgment and is inherently subjective [42]. Consequently, equity represents a more abstract and higher-order concept than equality. Westerners tend to prefer abstract principles and assume they are universally applicable [41]. Accordingly, Australian authors have concentrated on the governing principles of *equity* in education and predominantly used this term. In contrast, Chinese authors more frequently use *equality,* reflecting their focus on concrete substances in this research area.

These different perspectives of thinking between Western and Eastern cultures help distinguish the stance features of Chinese and Australian authors.

#### 4.2.3. Xin (integrity).

Although the tendency of Chinese authors to favor more assertive claims can be explained by the Confucian belief that verbal argumentation is unnecessary for knowledge construction [8,14], the Confucian concept of *xin* offers a new perspective for understanding this stance. *Xin* refers to loyalty and commitment. On the one hand, it entails being loyal to oneself by saying what one wants to say; on the other hand, it involves fulfilling one’s commitment [43]. Therefore, *xin* represents one of the most important moral principles in Chinese culture, emphasizing full commitment to one’s propositions. Excessive use of hedges by Chinese authors may lead readers to question their credibility, as hedging signals uncertainty. Chinese authors demonstrate a more assertive stance due to the full commitment they are expected to make in Chinese culture.

#### 4.2.4. We consciousness.

The concept of the person in Chinese culture is not merely an independent individual, “but involves a reciprocal relationship of linkage with another” [44]. Confucianism advocates the development of interrelationships among people, forming a web-like social structure that gradually encourages a person to “relinquish the private and individuated self (called *xiao wo*, 小我, literally meaning the small self)” and “embrace a larger collectivity to which one belongs as the operating self (called *da wo* 大我, the large self)” [45]. The boundaries of large selves can vary, reflecting the different collectivities with which one identifies, such as family members, friends, and associates, the community, the country, or even the world, which expand progressively through self-cultivation [45].

The *we* consciousness of the Chinese people, which incorporates others into the concept of the self, is also highlighted in the traditional Chinese notion of the unity of man and Heaven. The inseparable relationship between man and Heaven requires humans to “embody the laws of Heaven and to be responsible for it” [46]. This collectivist worldview gradually fosters a systemic and analytical perspective, shaping the Chinese understanding of the interrelationship among all things in the world and the principles governing their operation.

The Chinese *we* consciousness explains Chinese writers’ frequent use of the inclusive *we* as an engagement marker, along with their significantly fewer self-mentions compared to Australian authors. Chinese authors tend to perceive readers as part of the large self within the context of academic publications, emphasizing this collective perspective while downplaying the presence of an individuated or small self. However, Australian authors, shaped by their *I* consciousness and individualistic culture, reinforce their author stance through the frequent use of the exclusive *we* in their self-mention.

## 5. Conclusion and implications

This study examines author stance and engagement in research articles on educational equity by comparing Chinese and Australian authors, and reveals a substantially greater use of interactional MDMs by Chinese authors in all subcategories, except for self-mentions. The high frequencies of hedge markers and self-mentions are characteristic of English research articles, which tend to adopt a more cautious writing style that allows space for diverse opinions and maintains an appropriate balance between hedge markers and boosters. In contrast, Chinese authors often exhibit a more assertive and emotional style, demonstrated by a higher frequency of boosters and attitude markers. The frequency of engagement devices was also significantly higher among Chinese authors, primarily due to the frequent use of the inclusive *we.* Furthermore, this study adopts keyword analysis, enabling a close investigation of the collocates of statistically significant frequent words in the corpora and revealing the underlying cultural factors that contribute to these discursive features.

The research findings have important pedagogical implications for teaching academic writing. EAP (English for academic purposes) and ESP (English for specific purposes) teachers would benefit from emphasizing the importance of Chinese authors being less assertive and emotional by reducing the use of boosters and attitude markers, while encouraging greater use of self-mentions to strengthen author authority. Although the Chinese authors in this study employ significantly more hedges than their Australian counterparts, the ratio of hedges to boosters remains lower than that observed in Australian authors’ research articles. Therefore, it is necessary to draw novice L2 writers’ attention to achieving a proper balance between hedges and boosters and to teach them effective strategies for rhetorical construction that convey appropriate author stances in terms of assertiveness.

As cultural influences play a significant role in shaping author stances and engagement, EAP/ESP teachers should educate novice writers about discursive differences across cultures and explain the underlying causes of these variations to strengthen cross-cultural awareness. Teaching L2 academic writing should help writers learn both the vocabulary and syntactical features of the target language. More importantly, it should foster an understanding of the values and conventions within the target language’s culture, particularly within specific disciplinary fields.

It is recommended that Chinese authors approach their writing from the perspective of individual concepts and consider incorporating more collocates of *the X of equity* favored by Australian authors to complement the Chinese definition of educational equality. By doing so, Chinese authors can balance their studies’ focus between the concrete and the abstract aspects of the research topic. They may adopt a more balanced stance when presenting research findings, addressing both the attributes of the topic and its relationship with other issues across various contexts. This approach enables researchers to offer more practical and persuasive suggestions based on their findings.

EAP/ESP teachers should provide L2 writers with a broader range of writing strategies, enabling them to adapt their writing styles flexibly to meet the cognitive preferences of potential readers in other discourse communities and to clearly convey their intended messages. Such strategies can help reduce potential misunderstandings, allow authors to present the appropriate stance and engagement in their research articles, and improve the reception of their work by English-speaking audiences.

## 6. Limitations and further studies

This research is limited by its focus on studies examining author stance and engagement with a particular theme within a single discipline. Although the findings may not be generalizable to other disciplines, the focal study provides in-depth insights into the features of author stance and engagement, which are shaped by cultural influences to some extent. Similarly, the limited corpus size of this study restricts the generalizability of its findings to Chinese and Australian academic writing at large. It is plausible that sub-cultural groups within these broader communities may be shaped by distinct academic traditions, institutional practices, or disciplinary conventions and exhibit variations in stance and engagement features, despite sharing overarching Eastern or Western cultural influences.

While the present study analyzes the research articles as a whole, different units of analysis may reveal varying patterns of author stance and engagement features. Therefore, future research can benefit from comparative studies between L2 and English writers focusing on a particular section (e.g., the abstract) of research articles. Additionally, future research can examine author stances and engagement patterns between English research articles written by novice L2 writers (e.g., in China, Iran, Spain) and those by experienced L2 writers, potentially generating valuable pedagogical implications.

## Supporting information

S1 File Supporting information confirmed. (RAR)
